# Type 2 diabetes mellitus is more prevalent among patients with thyroid carcinoma and influences overall survival: a propensity score matching analysis

**DOI:** 10.18632/oncotarget.22179

**Published:** 2017-10-31

**Authors:** Tao Jiang, Guoliang Qiao, Xiao Zheng, Zhen Wen, Dongxue Zhang

**Affiliations:** ^1^ Department of Endocrinology, Beijing Shijitan Hospital, Capital Medical University, Beijing 100038, China; ^2^ Department of Medical Oncology, Beijing Key Laboratory for Therapeutic Cancer Vaccines, Capital Medical University Cancer Center, Beijing Shijitan Hospital, Capital Medical University, Beijing 100038, China

**Keywords:** T2DM, overall survival, PSM, TC

## Abstract

The relationship between Type 2 Diabetes Mellitus(T2DM) and cancer risk has been investigated for more than a decade. Many types of cancer were confirmed to be related with T2DM. The aim of this study is to identify the relationship between T2DM and the prevalence and long-term survivals of Thyroid Carcinoma(TC) using propensity score matching. In present study, 1658 thyroid nodule patients who were diagnosis in Beijing Shijitan hospital were divided into two groups: the TC group (N = 455, 27.4%), and the benign thyroid nodule(BTN) group (N = 1203, 73.6%). Propensity scores analyses were used to compare the overall survival (OS) and recurrence-free survival (RFS) between patients with or without T2DM. After propensity scores analyses, the prevalance of T2DM was significantly increased in the TC group compared with BTN group. Of the 455 TC patients, with T2DM in thyroid carcinoma was associated with increasing 1-, 3-, 5-year OS rates from 98.8, 76.5, and 70.9% to 99.7, 92.2, and 82.7%, respectively (P=0.017). While the 1-, 3-, and 5-year RFS rates in the group with T2DM were 92.3, 69.5, and 58.3%, which were significantly lower than those in the group without T2DM (97.6, 82.7, and 72.4%, P=0.009). After propensity scores analyses, with T2DM was significantly associated with increased risks of OS and RFS in the entire TC cohort.

## INTRODUCTION

Thyroid nodule(TN) is a common clinical endocrine disease, and the presence of thyroid nodule was about 4-7% in population worldwide [1, 2]. With the advancement of Computed tomography(CT), Magnetic resonance imaging(MRI), high-resolution ultrasound, isotope scanning and other medical imaging technologies, many hidden thyroid nodules can now be found [3, 4]. Studies showed that even thyroid solid nodules which were diagnosed as benign lesions still had 6% possibility of being malignant lesions when diagnosed by surgical pathology [5]. Therefore, active treatments should be conducted for suspected malignant thyroid nodules diagnosed by clinical and imaging examinations [6]. The most important reason is that thyroid carcinoma (TC) is becoming increasingly prevalent in recent years, which is account for 5∼15% of TN [7]. Although it has been reported that TC presents a relatively excellent prognosis previously [8], about 10% of the patients still die of this cancer [9].

Type 2 Diabetes Mellitus(T2DM) is one of the most rapidly increasing public health worldwide. The prevalence of T2DM is expected to an increase from 2.8% in 2000 to 4.4% in 2030, with the rate increasing rapaidly in developing countries than in developed ones [10]. The relationship between T2DM and cancer risk has been investigated for more than a decade. Studies showed that patients with T2DM had a higher risk of cancers involving the breast, endometrium, stomach, colorectum, liver, pancreas, urinary bladder, and non-Hodgkin’s lymphoma [11–15]. With respect to patients with TC, the results had not been consistent, due largely to the small number of incidence cases of thyroid carcinoma in any given study [16–18]. Patients with T2DM and thyroid carcinoma had an advanced tumor-node-metastasis stage at the time of diagnosis and an increased disease-specific mortality [19]. Nevertheless, the influence of T2DM on the prevalence and prognosis of TC is still unclear and controversial.

To address this issue, we conducted a retrospective cohort study to elucidate the relationship between T2DM and the prevalence and long-term survivals of TC using propensity score matching.

## RESULTS

### Propensity score matching with TC

1658 thyroid nodule patients who were diagnosis in Beijing Shijitan hospital were divided into two groups: the TC group (N = 455, 27.4%), and the BTN group (N = 1203, 73.6%). The comparisons of patients’ characteristics between these two groups in the entire cohort are illustrated in Table 1. Patients’ characteristics including age, sex, BMI, TSH level, TgAb levels, CT levels, nodule size and T2DM were significantly different between the two groups (all p <0.05). In the entire cohort, the prevalance of T2DM was significantly increased in the TC group compared with BTN group.

**Table 1:** Comparisons of patients’ characteristics between thyroid carcinoma(TC) and benign thyroid nodules(BTN) groups in the entire cohort

Variable	Thyroid carcinoma group(TC)	Benign thyroid nodules group(BTN)	P value
**case,n**	455	1203	
**age**	50.39±11.92	46.35±12.33	0.001
**Sex**			0.001
Female	379	843	
Male	76	360	
**BMI (kg/m2)**			0.001
<23	120	658	
>or=23	335	545	
**TSH levels (mU/L)**	6.84±5.32	4.55±3.42	0.001
**TPOAb levels (IU/mL)**	10.12±2.15	10.62±3.64	0.752
**TgAb levels (μg/L)**	35.51±13.32	28.71±13.07	0.001
**CA199 levels (U/ml)**	12.14±4.3	13.7±6.01	0.231
**CEA levels (ng/ml)**	3.12±1.04	2.85±1.08	0.157
**AFP levels (ng/ml)**	11.25±10.69	12.92±9.34	0.142
**CT levels (ng/L)**	23.6±6.25	17.1±5.43	0.001
**Nodule size**	2.634±1.15	1.365±2.06	0.043
**Lymph node metastases**	76	_	
**Distant metastases**	65	_	
**TNM staging**		_	
I-II	235	_	
III-IV	220	_	
**Hashimoto Thyroidtitis**			
Yes	245	737	0.315
No	210	566	
**Pathological type**		_	
papillary carcinoma	321	_	
follicular carcinoma	47	_	
undifferentiated carcinoma	35	_	
**medullary carcinoma**	52	_	
**T2DM**			0.001
Yes	87	112	
No	368	1138	

Body Mass Index(BMI), thyroid-stimulating hormone(TSH) levels, thyroid peroxidase antibody(TPOAb) levels, thyroglobulin antibody(TgAb) levels, alpha-fetoprotein(AFP), carcinoembryonic antigen(CEA), carbohydrate antigen 199(CA199) levels, Calcitonin(CT) levels, Type 2 Diabetes Mellitus(T2DM).

According to the variable of TC, propensity score matching analysis created 276 pairs of patients. Comparisons of patients’ characteristics between the TC and BTN groups in the propensity matched cohort are illustrated in Table 2. Apart from the variable of T2DM, all other variables were balanced between the two groups (all p >0.2). In the propensity matched cohort, the prevalance of T2DM was significant different between the TC and BTN groups (p = 0.027).

**Table 2:** Comparisons of patients’ characteristics between TC and BTN groups in the propensity matched cohort

Variable	Thyroid carcinoma group(TC)	Benign thyroid nodules group(BTN)	P value
**case,n**	276	276	
**age**	50.1±7.83	49.35±8.72	0.288
**Sex**			0.169
Female	214	200	
Male	62	76	
**BMI (kg/m2)**			0.281
<23	87	99	
>or=23	189	177	
**TSH levels (mU/L)**	5.12±3.67	4.89±3.76	0.467
**TPOAb levels (IU/mL)**	9.72±2.01	9.61±2.53	0.622
**TgAb levels (μg/L)**	30.43±9.88	29.47±10.65	0.272
**CA199 levels (U/ml)**	10.41±3.7	11.64±3.24	0.457
**CEA levels (ng/ml)**	2.97±2.52	2.87±2.58	0.266
**AFP levels (ng/ml)**	10.43±7.82	11.22±8.32	0.231
**CT levels (ng/L)**	21.73±4.51	20.25±3.52	0.572
**Nodule size**	2.433±1.32	2.162±1.85	0.462
**Hashimoto Thyroidtitis**			
Yes	145	150	0.671
No	131	126	
**T2DM**			0.027
Yes	67	46	
No	209	230	

Body Mass Index(BMI), thyroid-stimulating hormone(TSH) levels, thyroid peroxidase antibody(TPOAb) levels, thyroglobulin antibody(TgAb) levels, alpha-fetoprotein(AFP), carcinoembryonic antigen(CEA), carbohydrate antigen 199(CA199) levels, Calcitonin(CT) levels, Type 2 Diabetes Mellitus(T2DM).

### Propensity score matching with T2DM in patients with TC

Of 455 patients with TC, we divided them into two groups: the with T2DM group (N = 87, 19.1%), and the without T2DM group (N = 368, 80.9%). The comparisons of patients’ characteristics between these two groups in the entire cohort are illustrated in Table 3. Patients’ characteristics including sex, BMI, lymph node metastasis, TNM staging were significantly different between the two groups (all p <0.05).

**Table 3:** Comparisons of patients’ characteristics and pathological variables between with T2DM and without T2DM groups in the patients with TC

Characteristics	All TC patients (N=455)		P value
	With T2DM (N=87)	Without T2DM (N=368)	
**Gender**			0.038
male	20(22.98)	108(29.35)	
female	67(77.02)	260(70.65)	
**Age (years)**			0.705
<45	38(43.68)	169(45.92)	
≥45	49(56.32)	199(54.08)	
**BMI (kg/m2)**			0.001
<23	18(20.69)	168(45.65)	
≥23	69(79.31)	200(54.35)	
**Tumor size (cm)**			0.976
≤1	32(36.78)	136(36.96)	
>1	55(63.22)	232(63.03)	
**lymph node metastasis**			0.002
Yes	34(39.08)	94(25.54)	
No	53(60.92)	274(74.46)	
**TNM staging**			0.041
I-II	60(68.97)	289(79.35)	
III-IV	27(31.03)	76(20.65)	
**Distant metastases**			0.122
Yes	27(31.03)	85(23.09)	
No	60(68.97)	283(76.91)	
**Pathological type**			0.326
papillary carcinoma	49(56.32)	169(45.92)	
follicular carcinoma	14(16.09)	62(16.85)	
undifferentiated carcinoma	13(16.15)	74(20.11)	
medullary carcinoma	11(12.64)	63(17.12)	
**Hashimoto Thyroidtitis**			0.948
Yes	46(52.87)	172(46.74)	
No	41(47.13)	196(53.26)	

According to the variable of T2DM, propensity score matching analysis created 67 pairs of patients. Comparisons of patients’ characteristics between the with T2DM and without T2DM groups in the propensity matched cohort are illustrated in Table 4. All variables were balanced between the two groups (all p >0.2).

**Table 4:** Comparisons of patients’ characteristics and pathological variables between with T2DM and without T2DM groups in patients with TC in the propensity matched cohort

Characteristics	With T2DM (N=65)	Without T2DM (N=65)	P value
**Gender**			0.545
male	15(23.08)	18(27.69)	
female	50(76.92)	47(72.31)	
**Age (years)**			0.724
<45	30(46.15)	28(43.08)	
≥45	35(53.85)	37(56.92)	
**BMI (kg/m2)**			0.690
<23	16(24.62)	18(27.69)	
≥23	49(75.38)	47(72.31)	
**Tumor size (cm)**			0.218
≤1	26(40.00)	33(50.77)	
>1	39(60.00)	32(49.23)	
**lymph node metastasis**			0.850
Yes	21(32.31)	20(30.77)	
No	44(67.69)	45(69.23)	
**TNM staging**			0.560
I-II	45(69.23)	48(73.85)	
III-IV	20(30.77)	17(26.15)	
**Distant metastases**			0.840
Yes	17(26.15)	16(24.62)	
No	48(73.85)	49(75.38)	
**Pathological type**			0.942
papillary carcinoma	40(61.54)	38(58.46)	
follicular carcinoma	8(12.31)	10(15.38)	
undifferentiated carcinoma	10(15.38)	9(13.85)	
medullary carcinoma	7(10.77)	8(12.31)	
**Hashimoto Thyroidtitis**			0.861
Yes	31(47.69)	32(49.23)	
No	34(52.31)	33(50.77)	

### T2DM was an independent risk factor for prognosis of patients with TC

**Figure 1:**
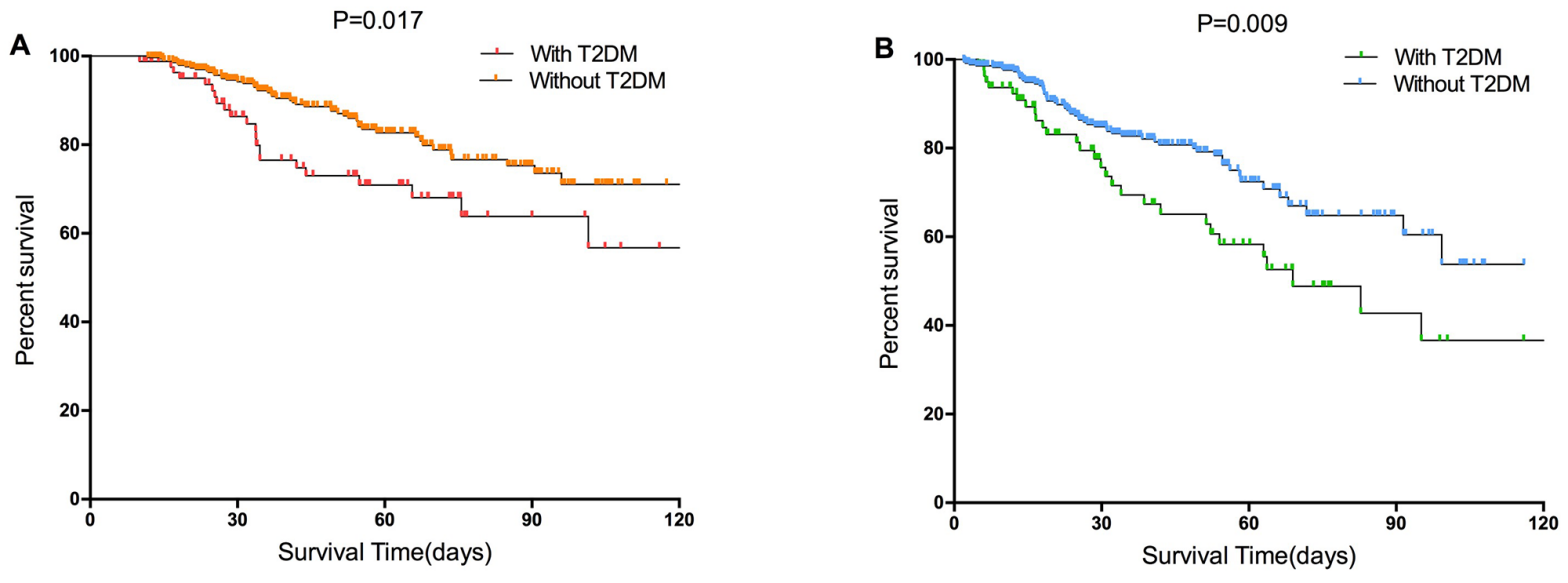
Survival curves of the with T2DM and without T2DM groups in the entire cohort **(A)** Overall survival (p=0.017 by log-rank test). **(B)** Recurrence-free survival (p=0.009 by log-rank test) in patients with TC.

Before propensity matching, with T2DM in thyroid carcinoma was associated with a increasing 1-, 3-, 5-year OS rates from 98.8, 76.5, and 70.9% to 99.7, 92.2, and 82.7%, respectively (P=0.017, Figure 1A). While the 1-, 3-, and 5-year RFS rates in the with T2DM and without T2DM groups were 92.3, 69.5, and 58.3%, and 97.6, 82.7, and 72.4%, respectively (P=0.009, Figure 1B). With T2DM was significantly associated with increased risks of OS and RFS in the entire TC cohort.

**Figure 2:**
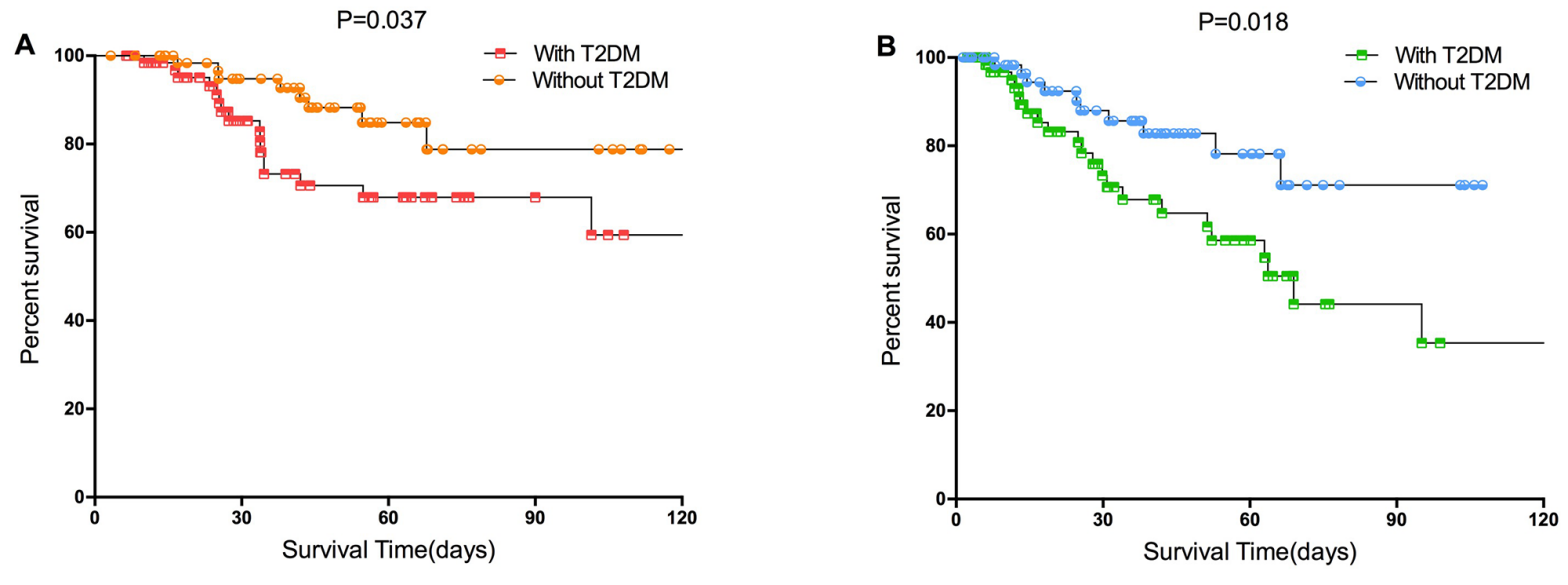
Survival curves of the with T2DM and without T2DM groups in the propensity matched cohort **(A)** Overall survival (p=0.037 by log-rank test). **(B)** Recurrence-free survival (p=0.018 by log-rank test) in patients with TC.

After propensity matching, with T2DM in thyroid carcinoma was associated with a increasing 1-, 3-, 5-year OS rates from 98.4, 73.3, and 67.9% to 100, 94.8, and 84.9%, respectively (P=0.037, Figure 2A). While the 1-, 3-, and 5-year RFS rates in the with T2DM and without T2DM groups were 93.1, 67.8, and 58.6%, and 98.3, 85.7, and 78.2%, respectively (P=0.018, Figure 2B). With T2DM was significantly associated with increased risks of OS and RFS in the TC propensity matching cohort.

## DISCUSSION

The dramatically increasing incidence of thyroid carcinoma might be partly attributed to detection bias due to increasing screening by neck ultrasound; but it cannot be fully explained by increased medical surveillance or improved detection methods alone [20]. Moreover, increased prevalence of large thyroid tumors (>5 cm) suggested that other contributing factors may be involved in the development of thyroid carcinoma which needs further elucidation [21]. Here we address the possible role of T2DM.

Several studies had showed a higher prevalence of thyroid disorders among diabetics compared with the general population, and the potential roles of metabolic syndrome, obesity, and diabetes as precipitating factors in cancer development suggested that diabetes might play a role in thyroid carcinoma risk [22, 23]. In this study, our results showed that the prevalance of T2DM was significantly increased in the TC group compared with BTN group. The critical point was that the control group in this study was BTN group, rather than general population. Moreover, after propensity score matching analysis, T2DM was still an independent risk factor for TC.

The potential mechanism of T2DM on TC was still unclear. Firstly, in laboratories, thyroid carcinoma cell lines are grown using a medium containing TSH, which is susceptible to disruption in thyroid hormone homeostasis [24, 25]. Secondly, elevated circulating insulin levels in T2DM with insulin resistance may influence thyroid carcinoma risk mediated by insulin receptors overexpressed by cancer cells, suggesting that insulin may play a role in thyroid carcinogenesis. IGF-1, a well-known pathway with an affinity for insulin, is also critical to cell proliferation and apoptosis and has been shown to be related to various types of cancers [26]. Thirdly, Chronic metabolic disturbances included aberrations in the insulin-like growth factor pathway, also affected steroid hormone metabolism suggesting that this pathway may also be involved [27, 28].

Although many studies investigated the relationship of T2DM with TC, few researches concentrated on the prognostic significance of T2DM on patients with TC [19]. The present study showed that T2DM was significantly associated with increased risks of OS and RFS in the TC cohort both before and after propensity score matching analysis. More importantly, twice propensity score matchings were performed in this study, which made the comparison between groups with and without T2DM more reasonable.

This study has several limitations. First, this is a retrospective cohort study but not a randomized controlled trial. However, for the research question at hand, it is nearly impossible and to perform a randomized trial. Furthermore, A cohort study applying propensity score matching, as done in the present research, represents the best-suited study design. Second, in this single-center study, no stratified analysis was performed according to the variable of pathological type. Now we are in the process of data collection and we would perform subgroups analysis comparing well-differentiated thyroid carcinoma with others. Finally, external validation from independent study groups is necessary to confirmed the conclusions in this study.

In summary, using propensity score matching analyses, our present study showed that the prevalance of T2DM was significantly increased in the TC group compared with BTN group. T2DM was not significantly associated with RFS and OS for patients with TC.

## MATERIALS AND METHODS

### Patient selection

This was a retrospective study, in which we collected the clinical data of 1658 thyroid nodule patients with postoperative pathological results in Beijing Shijitan hospital between January 2010 to December 2015, which included 455 patients with thyroid carcinoma and 1203 benign thyroid nodule(BTN).

Inclusion criteria: (1) Patients who are between 15 to 85 years old. Gender is not limited; (2) Patients with thyroid nodule who have accepted surgical treatment in our hospital between January 2010 to December 2015; (3) Patients who have postoperative pathologic results; (4) Patients without other tumor and whose function of heart, lung, liver, kidney and blood coagulation are normal; (5) Patients whose information are complete; (6) Women patients who are not pregnant or lactant.

### Diagnosis of TC

Pathological data: Collecting lesion number, maximum diameter, pathological type, lymph node metastasis, immunohistochemical and gene mutation of thyroid nodules. The total thyroid was submitted for microscopic examination and all slides were further evaluated using deeper sections. Pathological tissue processing: Intraoperative thyroid tissue specimens were made into frozen section with TT22-HQP-101 type constant cold box frozen section machine made by Germany Leica company; Postoperative thyroid tissue specimens were made into paraffin section with RM2235 paraffin section machine made by Germany Leica company. Two experienced pathology doctors diagnosed pathological type of TN according to the 2004 WHO diagnostic standard in Pathology and Genetics of Endocrine Organ Tumor [6, 29].

### Diagnosis of T2DM

Diagnosis of diabetes mellitus was based on the 1999 World Health Organization criteria [30]. Patients who were found to have a fasting blood sugar level between 5.6 and 6.1 mmol/L were defined as having impaired fasting glucose and were referred for confirmation of their diabetes status. Those with a fasting blood sugar level of at least 7.0 mmol/L or those diagnosed with Type 2 diabetes before entering the hospital were defined as having Type 2 diabetes and were referred for further diabetic care. Patients with prior diagnosis of Type 1 diabetes were excluded from the study.

### Follow-up

The follow-up ended on July 2016; the median follow-up duration was 58.2 months (range from 3.8 to 116.8 months). Neck ultrasonography was carried out once every 3 months in the first two years after surgery, and then once every 6 months thereafter. The diagnostic criteria for TC recurrence were the same as used for the initial diagnosis.

### Propensity score matching

Firstly, patients in the TN and TC groups were matched using the propensity score method as described by Rubin and Rosenbaum [31, 32], which was carried out using SPSS.22.0 software. The propensity score for an individual was calculated given the covariates of age, sex, Body Mass Index(BMI), thyroid-stimulating hormone(TSH) levels, thyroid peroxidase antibody(TPOAb) levels, thyroglobulin antibody(TgAb) levels, alpha-fetoprotein(AFP), carcinoembryonic antigen(CEA), carbohydrate antigen 199(CA199) levels, Calcitonin(CT) levels, nodule size, hashimoto thyroidtitis and T2DM. This method consisted of ordering the case and control subjects, then selecting the first case subject and finding the control subject with the closest propensity score. Both subjects were then removed from consideration for matching and the next case subject was selected [33]. We used the forward procedure which started out with just the intercept and sequentially added the effect that most improved the fit. Variables were included up to a limit of a monotonized p-to-enter value of <0.2. Thereafter, we applied 1:1 nearest neighbor matching without replacement in order to ensure that conditional bias was minimized. The nearest neighbor matching was based on a greedy matching algorithm, which matched each unit in the treatment group to a unit in the control group that had the closest propensity score. For each patient of TC, a patient in BTN with a minimum in distance of propensity scores was matched. We tested multiple caliper widths. The appropriateness of matching was assessed by comparing the standardized differences in covariate means for continuous and dichotomous variables for the matched and unmatched samples.

### Statistical analysis

Statistical analyses were carried out using the IBM SPSS Statistics 22.0 (SPSS Inc., Armonk, NY, USA). Continuous variables were expressed as mean±standard deviation (SD) or median (range). Categorical variables were reported as number (n) or proportion. The Student’s t test was used for comparisons of continuous variables when applicable. Otherwise, the Mann-Whitney U test was applied. Categorical variables were compared with the Chi square test with the Yates correction or the Fisher’s exact test, as appropriate. The overall survival(OS) and recurrence free survival(RFS) rates were compared between the with T2DM and without T2DM groups before and after propensity matching using the Kaplan-Meier curves generated by the log-rank test. p values <0.05 were considered statistically significant.

## Abbreviations

T2DM: Type 2 diabetes mellitus
TC: Thyroid carcinoma
OS: Overall survival
RFS: Recurrence-free survival
TN: Thyroid nodule
CT: Computed tomography
MRI: Magnetic resonance imaging
